# Identification of Immune-Related Genes for Establishment of Prognostic Index in Hepatocellular Carcinoma

**DOI:** 10.3389/fcell.2021.760079

**Published:** 2021-11-02

**Authors:** Yinfang Li, Ling Zou, Xuejun Liu, Judong Luo, Hui Liu

**Affiliations:** ^1^Aliyun School of Big Data, Changzhou University, Changzhou, China; ^2^School of Computer Science and Technology, Nanjing Tech University, Nanjing, China; ^3^Department of Radiotherapy, The Affiliated Changzhou No. 2 People's Hospital of Nanjing Medical University, Changzhou, China

**Keywords:** hepatocellular carcinoma, immune related genes, prognostic biomarker, immune infiltration level, tumor microenvironment

## Abstract

**Background:** Immune checkpoint inhibitor (ICI) therapy has been proved to be a promising therapy to many types of solid tumors. However, effective biomarker for estimating the response to ICI therapy and prognosis of hepatocellular carcinoma (HCC) patients remains underexplored. The aim of this study is to build a novel immune-related prognostic index based on transcriptomic profiles.

**Methods:** Weighted gene co-expression network analysis (WGCNA) was conducted to identify immune-related hub genes that are differentially expressed in HCC cohorts. Next, univariate Cox regression analysis and least absolute shrinkage and selection operator (LASSO) analysis were used to detect hub genes associated to overall survival (OS). To validate the immune-related prognostic index, univariate and multivariate Cox regression analysis were performed. CIBERSORT and ESTIMATE were used to explore the tumor microenvironment and immune infiltration level.

**Results:** The differential expression analysis detected a total of 148 immune-related genes, among which 25 genes were identified to be markedly related to overall survival in HCC patients. LASSO analysis yielded 10 genes used to construct the immune-related gene prognostic index (IRGPI), by which a risk score is computed to estimate low vs. high risk indicating the response to ICI therapy and prognosis. Further analysis confirmed that this immune-related prognostic index is an effective indicator to immune infiltration level, response to ICI treatment and OS. The IRGPI low-risk patients had better overall survival (OS) than IRGPI high-risk patients on two independent cohorts. Moreover, we found that IRGPI high-risk group was correlated with high TP53 mutation rate, immune-suppressing tumor microenvironment, and these patients acquired less benefit from ICI therapy. In contrast, IRGPI-low risk group was associated with low TP53 and PIK3CA mutation rate, high infiltration of naive B cells and T cells, and these patients gained relatively more benefit from ICI therapy.

## 1. Introduction

Liver cancer remains a global health challenge, with an estimated incidence of more than 1 million cases by 2025 (Llovet et al., 2021) around the world. Hepatocellular carcinoma (HCC) is the most common form of liver cancer and accounts for 90% cases, and its increasing mortality rate is receiving growing concern. Conventional treatment, such as surgery, radiotherapy, and chemotherapy, do not significantly prolong overall survival (OS) of HCC patients (Ghouri et al., 2017).

Immunotherapy is emerged as an effective therapy in the field of cancer treatment in recent years, and among them the most impressive is immune checkpoint blockade (Mellman et al., 2011). Its clinical advantage including but not limited to continuous anti-tumor immune response with relatively weak side effect, low recurrence rate, and even complete remission for some advanced cancers (Khalil et al., 2016). As a result, immune checkpoint blockade has been approved for first-line therapy of some cancers.

For HCC, ICI has shown strong anti-tumor activity in a portion of patients (Khalil et al., 2016; Hou et al., 2020). Especially, the combination of the anti-PDL1 antibody atezolizumab and the VEGF-neutralizing antibody bevacizumab has become the standard of care as a first-line therapy for HCC (Sangro et al., 2021). However, the fraction of HCC patients that benefit from immunotherapy remains very limited, while other immunotherapy such as adoptive T-cell transfer, vaccination, or virotherapy have not yet demonstrated consistent clinical activity. As the factors that influence ICI efficacy are multifaceted, such as the immune microenvironment (TME) and PD-L1 level, the establishment of gold-standard biomarker for immunotherapy benefit is challenging (Nishino et al., 2017).

In this paper, we resort to transcriptomic data and clinical outcome to establish an immune-related gene prognostic index (IRGPI) of HCC, by exploiting weighted gene co-expression network analysis (WGCNA) (Langfelder and Horvath, 2008). Among 655 differentially expressed genes related to immunity, 148 hub genes were identified by WGCNA. Next, we identified 25 genes significantly related to overall survival by univariate Cox regression analysis, and selected 10 genes by LASSO analysis to construct the immune-related prognostic index (IRGPI), a quantitative score indicative of low vs. high risk of prognosis. Further analysis confirmed that the IRGPI is closely associated to immune microenvironment, response to ICI treatment and OS. In particular, the low-risk patients had better overall survival (OS) than high-risk patients, for both TCGA and GEO cohorts. Moreover, high-risk group was correlated with high TP53 mutation rate, immune-suppressing tumor microenvironment, and these patients acquired less benefit from ICI therapy. In contrast, low-risk group was associated with low TP53 and PIK3CA mutation rate, high infiltration of naive B cells and T cells, and these patients gained relatively more benefit from ICI therapy. Finally, we also validated IRGPI outperformed routine biomarkers used to screen patients who can benefit from immunotherapy. The results suggested that IRGPI was a promising prognostic biomarker for patients.

## 2. Materials and Methods

### 2.1. Data Resource and Preprocessing

The cohort for IRGPI establishment was obtained from TCGA (TCGA cohort), which includes 424 samples (374 cancer samples and 50 para-cancer samples). We utilized the UCSC Xena browser (Goldman et al., 2020) to download the RNA-seq data (FPKM normalized). The matched somatic mutation data and clinical outcome of the HCC patients were also obtained.

An independent cohort was obtained from the GSE14520 to verify the effectiveness of the IRGPI risk score. The validation cohort includes 221 HCC patients, and the transcriptomic data and clinical outcome were obtained from GEO database (Edgar et al., 2002). To further verify the predictive performance of the IRGPI on ICI therapy, we employed GSE140901 dataset, which has the gene expression profiles and clinical outcome of HCC patients who received anti-PD-L1 therapy.

The immune-related genes were downloaded from ImmPort database (Bhattacharya et al., 2014) and InnateDB database (Breuer et al., 2013). After removal of duplicate genes, the ImmPort database contained 1,811 immune genes and the InnateDB database contained 1,226 immune genes.

Associations between protein-coding genes (mRNA) and transcriptional factors (TFs), miRNA and lncRNA were downloaded from TRRUST v2 (Han et al., 2018), TargetScan (Lewis et al., 2005), miRDB (Wong and Wang, 2015), miRTarBase (Chou et al., 2018), and miRcode databases (Jeggari et al., 2012), respectively.

### 2.2. Differential Expression and Enrichment Analysis

The *limma* package (version 3.44.3) (Ritchie et al., 2015) was used to identify the differentially expressed mRNA. The log2 fold change>1 and FDR <0.05 were used as the criteria to screen differentially expressed genes (DEGs). Enrichment analysis on Gene Ontology (GO) and KEGG pathways were conducted using the *clusterProflier* R package (Yu et al., 2012). *Treemap* R package (Baehrecke et al., 2004) were chosen for visualization of significant functional annotations and pathways.

### 2.3. Identification of Hub Genes by WGCNA

Weighted gene co-expression network analysis (WGCNA) was performed to identify hub genes. First, the similarity matrix was constructed by using the expression data by calculating the Pearson correlation coefficient between gene pairs. Next, the similarity matrix was transformed into an adjacency matrix with a network type of signed and then transformed into a topological matrix with the topological overlap measure (TOM) describing the degree of association between genes. 1-TOM was used as the distance to cluster the genes, and then the dynamic pruning tree was built to identify the modules. Finally, we identified the modules by setting the merging threshold function at 0.25.

The modules with significantly different expression patterns between tumor and normal tissues were chosen for downstream analysis. We took the intersection between the modules and differentially expressed immune-related genes. To assess the potential biologic functions and involved pathways of the differentially expressed immune-related genes, gene set enrichment analysis (GSEA) was conducted using *clusterProfiler* R package.

### 2.4. Construction of Immune-Related Gene Prognostic Index

To develop a prognostic index, univariate Cox regression analysis and least absolute shrinkage and selection operator (LASSO) were conducted to assess the associations between immune-related genes expressions and overall survival (OS). Cox and LASSO regression were carried out using *survival* R package (Therneau and Lumley, 2014) and *glmnet* R package (Hastie and Qian, 2014).

The IRGPI risk score was computed using each gene expression level multiplied by its linear regression coefficient obtained from the univariate Cox regression. According to the median risk score, patients were assigned to high-risk and low-risk groups. The Kaplan-Meier survival analysis was performed to compare overall survival between high-risk and low-risk groups. The receiver operating characteristic (ROC) curves were plotted by *survival* R package. To validate the independent prognostic value of IRGPI, univariate and multivariate Cox regression analysis was performed on IRGPI risk score and other clinicopathologic feature.

### 2.5. Construction of Regulatory Networks

First, we identified the differentially expressed long non-coding RNAs (DElnRNAs) and miRNAs (DEmiRNAs) by setting the standards that log2 fold change>1 and FDR <0.05. Next, multiple miRNA target databases, including TargetScan, miRTarBase, and miRDB, were used to seek DEmiRNAs targeting the hub immune-related DEGs. The miRcode database was used to find lncRNA-miRNA associations. The TRRUST database version 2 was used to search the TFs regulating the hub immune-related DEGs. Finally, Cytoscape was used to visualize the regulatory network containing the miRNA-mRNA, lncRNA-miRNA, and TFs-mRNA associations.

### 2.6. TME and ICI Efficacy Compared Between IRGPI Groups

To explore the immune microenvironment of HCC, the transcriptomic data were imported into CIBERSORT (Newman et al., 2015)to compute the proportion of 22 types of immune cells. We compared the relative proportions of these immune cell types between two IRGPI groups. The immune infiltrating cells and tumor purity were assessed by ESTIMATE tool (Yoshihara et al., 2013). Besides, we explored the correlation between canonical immune subtypes and IRGPI groups.

Somatic mutation landscape was built in two IRGPI groups by using the *Maftools* R package (Mayakonda et al., 2018). Correlation analysis was performed between IRGPI risk score and conventional immunotherapy biomarkers, including programmed death receptor ligand-1/2 (PD-L1/2), programmed cell death protein-1 (PD-1), cytotoxic T lymphocyte associated antigen-4 (CTLA-4), cytolytic activity (CYT), and tumor mutation burden (TMB).

For estimation of the potential clinical efficacy of ICI treatment in different IRGPI groups, we calculated tumor immune dysfunction and exclusion (TIDE) scores (Jiang et al., 2018) in different IRGPI groups. Tumor inflammation signature (TIS) is an 18-gene signature that reflects an ongoing adaptive Th1 and cytotoxic CD8+ T cell response and shows promising results in predicting response to anti-PD-1/PD-L1 agents (Ayers et al., 2017). IRGPI risk score was also used to predict the overall survival of patients received anti-PD-L1 agents. ROC curves and AUC value were used to estimate the performance of IRGPI risk score. Performance comparison was also performed between IRGPI risk score and TIDE and TIS scores.

### 2.7. Statistical Analysis

Independent *t*-test was performed to compare continuous variables between two groups. Categorical data were tested using the chi-square test. TIDE score between groups was compared by the WilCoxon test. Univariate survival analysis was performed by Kaplan-Meier survival analysis with the log-rank test. A two-sided *p*-value < 0.05 was considered significant.

## 3. Results

### 3.1. WGCNA Analysis Identified Immune-Related Hub DEGs

The differential expression analysis on 374 tumors and 50 normal TCGA samples yielded a total of 6,209 differentially expressed genes (DEGs), in which 5,391 genes were up-regulated and 818 genes were down-regulated. By intersecting DEGs with the immune-related genes collected from ImmPort and InnateDB databases, 655 differentially expressed immune-related genes were obtained, in which 460 genes were up-regulated and 195 were down-regulated in tumor samples compared with normal samples.

**Figure 1:**
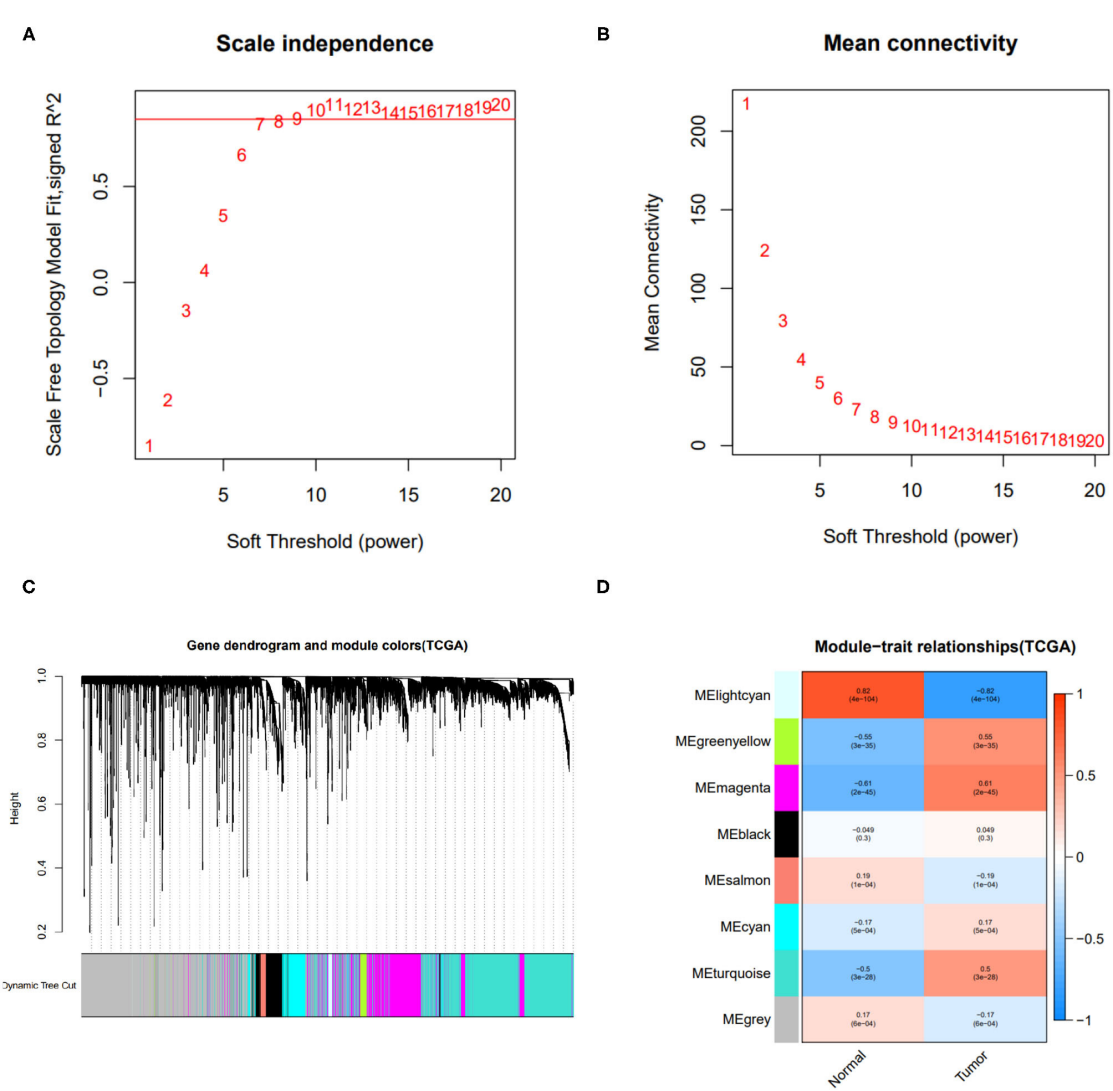
Results of weighted gene co-expression network analysis (WGCNA). **(A)** The scale-free fit index of various soft-thresholding powers. **(B)** Mean connectivity of various soft-thresholding powers. **(C)** A dendrogram of the differentially expressed genes clustered based on different metrics. **(D)** Heatmap of associations between module eigengenes of normal and tumor tissues.

To obtain the immune-related hub genes, WGCNA analysis was carried out. As shown in Figures 1A,B, the logarithm log(*k*) of the node with connectivity *k* was negatively correlated with the logarithm log[P(k)] of the probability of the node, and the correlation coefficient was >0.85. The optimal soft-thresholding power was set to 10. Eight modules were identified based on the average linkage hierarchical clustering and the optimal soft-thresholding power (Figures 1C,D). According to the Pearson correlation coefficient between a module and sample feature, we found green, yellow, and magenta modules were closely correlated with HCC tumors, the genes in these two modules were selected for further analysis.

Taking the intersection between the genes included in the two WGCNA-derived modules and the lists of differentially expressed immune-related genes, 148 immune-related hub genes (IRGs) were obtained, of which 122 genes were up-regulated and 26 were down-regulated.

### 3.2. Treemaps of Enriched Functional Annotations and Pathways

**Figure 2:**
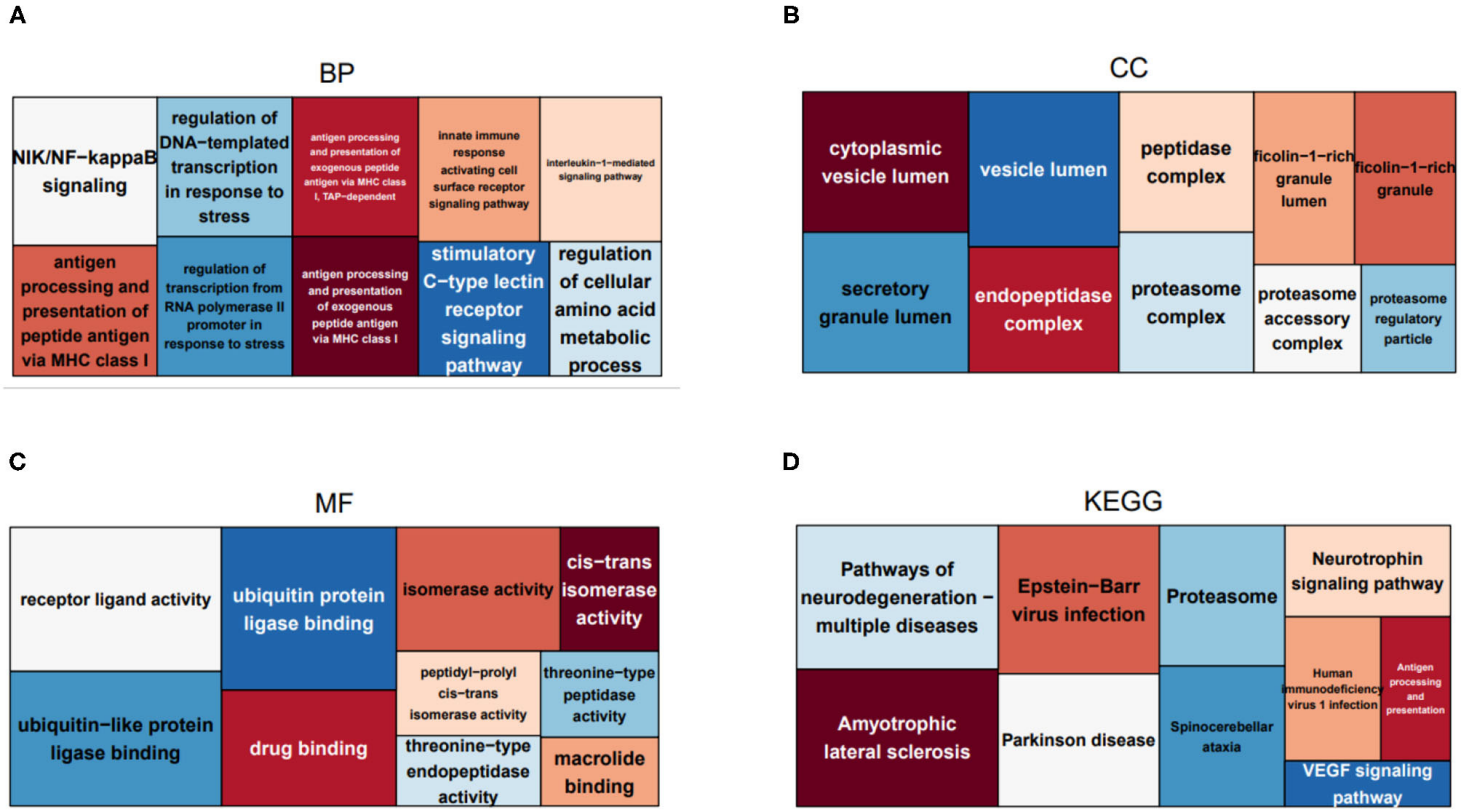
Treemaps of GO and KEGG enrichment analysis, in which rectangle size is proportional to the statistical significance of functional annotations and pathways. **(A–C)** Treemaps of the enrichment analysis on GO biological process, cellular component, and molecular function, respectively. **(D)** Treemaps of enrichment analysis on KEGG pathways.

To uncover the role of the immune-related hub genes in the pathogenesis of HCC, GO, and KEGG enrichment analysis was carried out. The top 10 significantly enriched GO terms and KEGG pathways for IRGs are shown in Figure 2. As for cellular component, the set of IRGs are significantly enriched in the regulation of response to biotic stimulus, regulation of innate immune response, fc receptor signaling pathway, and regulation of morphogenesis of an epithelium. As for cellular component, they are significantly enriched in cytoplasmic vesicle lumen, secretory granule lumen, and peptidase complex. For molecular function, they are significantly enriched in receptor ligand activity signaling receptor activator activity and ubiquitin-like protein ligase binding. As for KEGG pathway analysis, they are significantly enriched in Epstein-Barr virus infection, pathways of neurodegeneration-multiple diseases and amyotrophic lateral sclerosis.

### 3.3. IRGPI Risk Score Is Predictive of Overall Survival

The screened 148 immune-related hub genes were further evaluated by univariate Cox regression. As a result, 25 genes showed statistical significance to overall survival on TCGA cohort. Next, LASSO regression was performed to further narrow the scope of OS-related hub genes, and finally 10 genes were selected for the establishment of prognostic index. Specifically, the IRGPI risk score was computed using gene expression level multiplied by the weights of the 10 genes. The weights are shown in Table 1.

**Table 1:** The 10 immune-related hub genes used to compute IRGPI risk score.

**Gene**	**Coef**	**HR**	**HR.95L**	**HR.95H**	***p*-value**
CARS1	0.143645	1.68498	1.24329	2.283584	0.000768
CBS	−0.10811	0.656049	0.477454	0.901449	0.009325
CISD1	0.088366	1.631699	1.196967	2.224324	0.001953
GCLM	0.132237	1.445134	1.193399	1.749971	0.000163
SAT1	−0.06677	0.746933	0.576198	0.968259	0.027555
SLC7A11	0.086376	1.466116	1.232625	1.743836	1.54E-05
ACACA	0.116986	1.61311	1.228934	2.117383	0.00057
KEAP1	0.08436	1.54419	1.04808	2.275135	0.027987
SLC1A5	0.096401	1.363949	1.215323	1.530752	1.34E-07
G6PD	0.112358	1.415541	1.264641	1.584447	1.52E-09

**Figure 3:**
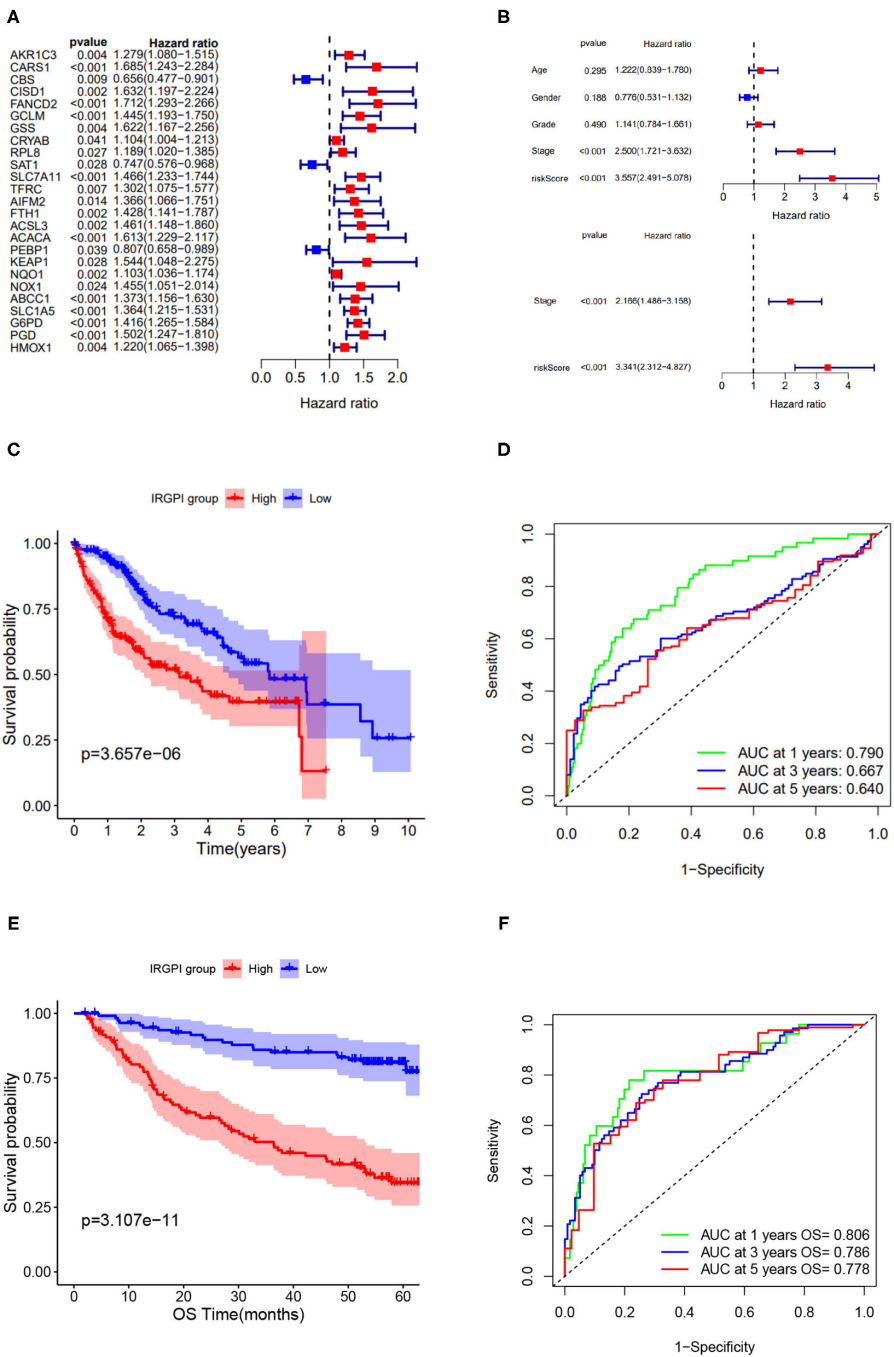
Screening of immune-related prognostic genes *via* Cox and LASSO regression and survival analysis of IRGPI low- and high-risk groups. **(A)** Univariate Cox regression of 25 immune-related hub genes. **(B)** Univariate and multivariate Cox regression analysis on IRGPI and other clinicopathologic variables. **(C)** Kaplan-Meier curve of the survival analysis between IRGPI groups on TCGA cohort. **(D)** ROC curve and AUC values of IRGPI risk score in predicting 1-, 3-, and 5-year OS on TCGA cohort. **(E)** Kaplan-Meier curve of the survival analysis between IRGPI groups on GSE14520 cohort. **(F)** ROC curve and AUC values of IRGPI risk score in predicting 1-, 3-, and 5-year OS on GSE14520 cohort.

The univariate Cox regression analysis was performed on IRGPI risk score and other clinicopathologic feature (Figure 3A), and verified that the IRGPI risk score and clinical stage were statistically significant factors with the prognosis of HCC (Figure 3B). Moreover, multivariate Cox regression analysis confirmed that IRGPI was an independent prognostic factor after adjusted for other clinicopathologic factors (Figure 3B).

Taking the median IRGPI score as the cutoff value to partition the TCGA cohort into low- and high- risk groups, the IRGPI low-risk patients showed obviously better OS than IRGPI high-risk patients (*p* = 0.001, log-rank test) (Figure 3C). The ROCAUC of the IRGPI prognosis model reach 0.790 for 1-year OS, 0.667 for 3-year OS, and 0.640 for 5-year OS (Figure 3D).

Furthermore, to evaluate the generalization of IRGPI in prognosis, we conducted survival analysis on validation cohort (*n* = 221). As showed in Figure 3E, the two IRGPI groups differed significantly in OS, and the IRGPI low-risk group had better prognosis than the IRGPI high-risk group (p = 0.0001, log-rank test). The ROCAUC of the prognostic model on validation cohort reach 0.806 for 1-year OS, 0.786 for 3-year OS, and 0.778 for 5-year OS (Figure 3F). Of note, the result on the validation cohort is consistent with that on the TCGA cohort, suggesting that the IRGPI could be a promising indicator for prognosis of HCC patients.

### 3.4. Regulatory Network of Immune-Related Hub Genes

**Figure 4:**
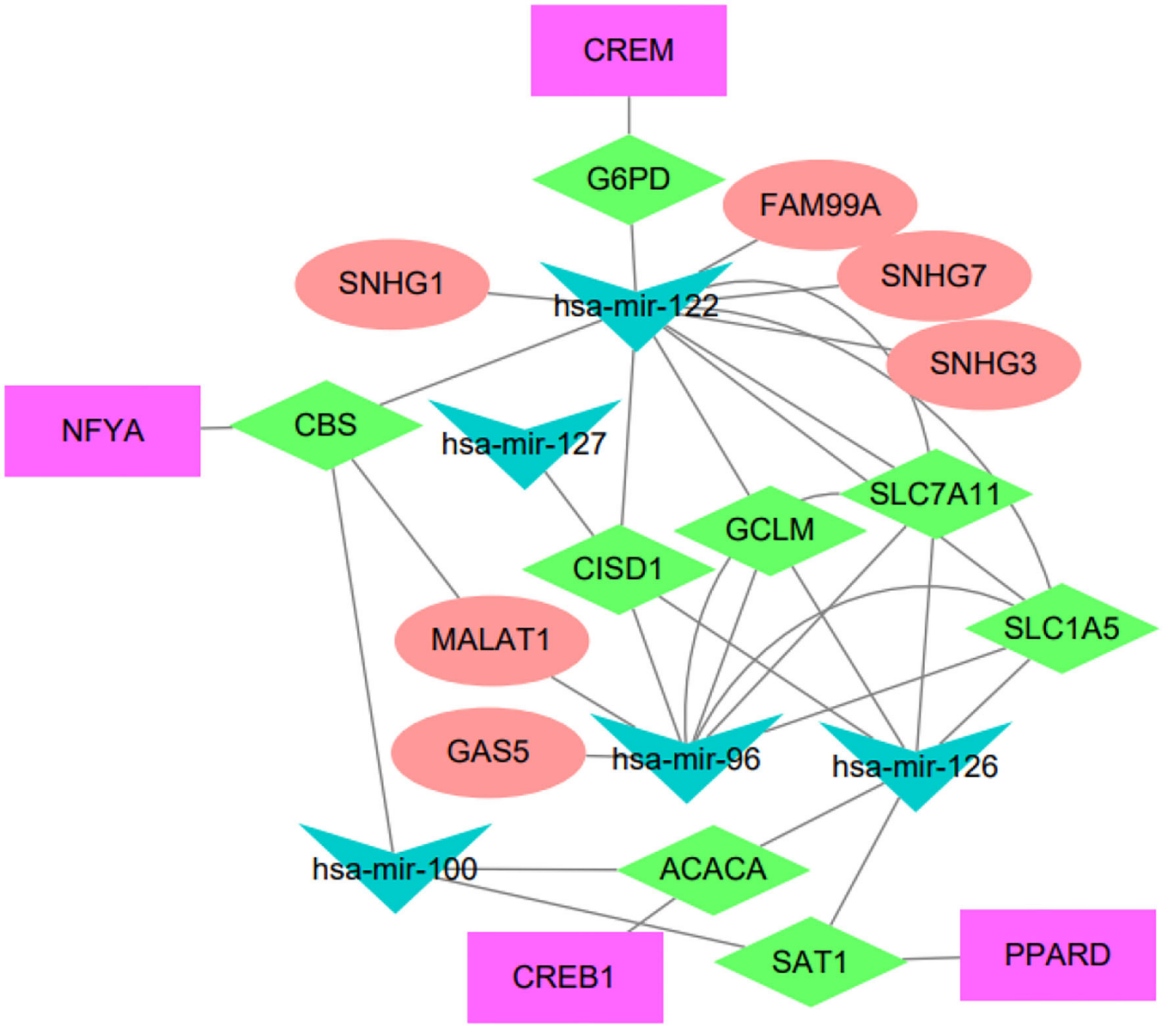
Regulatory network of the IRGPI-related genes. The network summarizes regulation relationships between transcription factors (purple rectangles), mRNAs (green diamonds), miRNAs (blue triangles), and lncRNAs (pink eclipse).

The regulatory network acts as an important role in tumorigenesis and development of HCC, we attempted to elucidate the endogenous regulatory mechanism of the immune-related hub genes. We built a regulatory network composed of mRNAs, miRNAs, lncRNAs, and transcription factors (TFs). To be specific, the regulatory network included 5 miRNAs, 6 lncRNAs, and 4 TFs associated to eight out of the immune-related hub genes, as shown in Figure 4.

We have conducted extensive literature study to verify these regulatory relationships. For example, the lncRNA FAM99A has been found to be up-regulated in HCC and closely related to clinical prognosis (Sun et al., 2020). There is increasing evidence that lncRNA GAS5 acts as a tumor suppressor, which is downregulated in certain tumor tissues and combined with miRNA to regulate related signaling pathways (Cheng et al., 2018). The lncRNA MALAT1 has been reported to be associated with diabetes-induced microvascular dysfunction, activate p38/MAPK signaling and regulate retinal endothelial cell function under diabetic condition (Liu et al., 2014). Another study indicated that MALAT1 regulated hyperglycemia induced inflammatory process in endothelial cells (Puthanveetil et al., 2015). The NFYA TF has been reported to be up-regulated in HCC and associated to tumors with mutant p53 (Bezzecchi et al., 2020). The CREB1 is a key transcription factor that mediates transcriptional responses to a variety of growth factors. CREB1 has been reported to be related with metastasis, tumor stage and poor outcome in gastric cancer (Wang et al., 2015), and the knockdown of CREB1 could inhibit liver cancer cell migration (Yang et al., 2013). PPARD has been reported to accelerate colorectal tumorigenesis, progression, and invasion (Liu et al., 2019), it could effectively predict the prognosis of HCC patients as an independent prognostic signature (Sun et al., 2021). It has been reported that hsa-miR-122-5p levels are connected with cholesterol levels in a viral hepatitis-free human population and associate with fatty liver and lipoprotein metabolism (Raitoharju et al., 2016).

### 3.5. TME and Somatic Mutations Differ in Two IRGPI Groups

**Figure 5:**
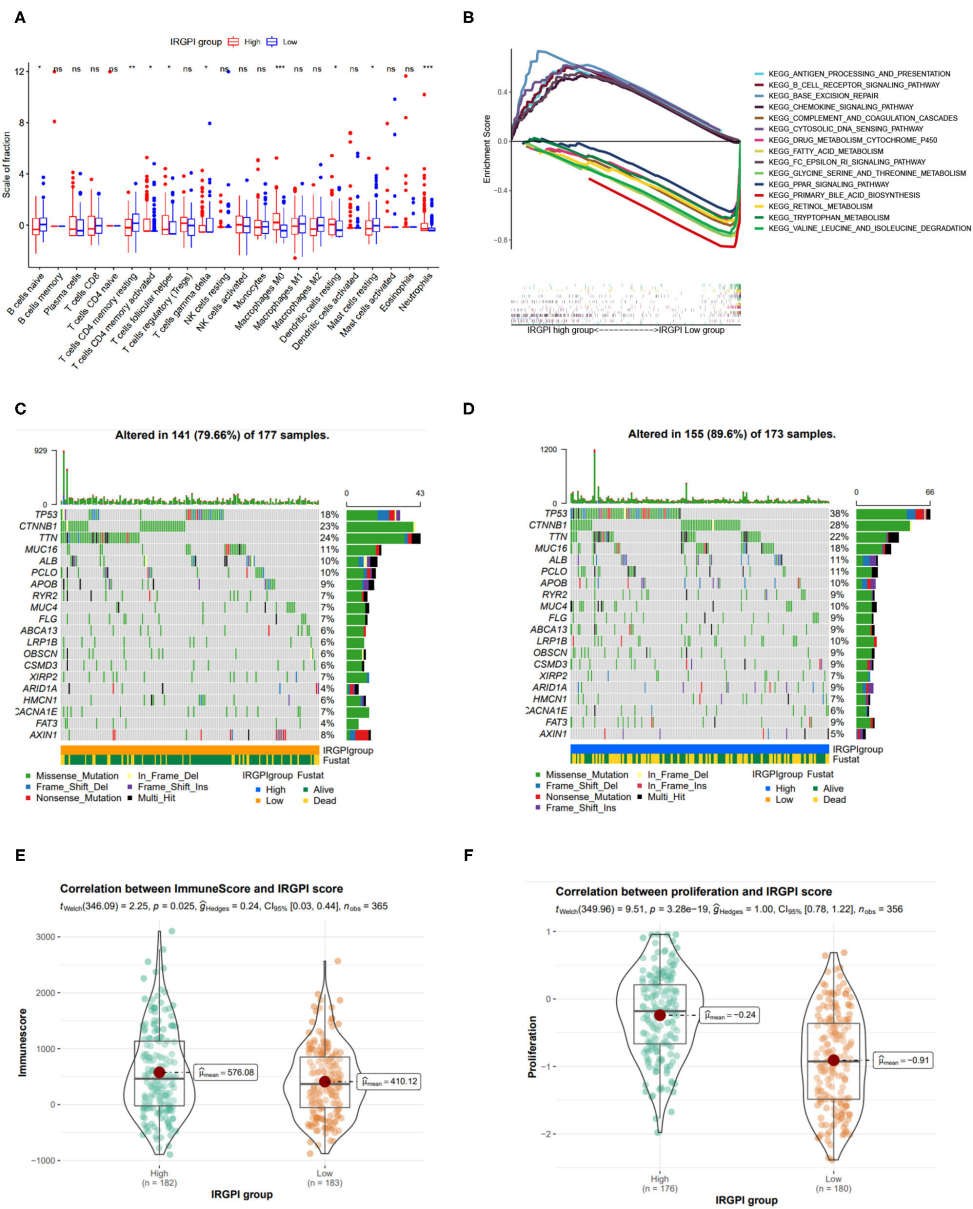
Tumor immune environment, somatic mutation landscape, and immune scores between IRGPI low- and high-risk groups. **(A)** Comparison of tumor immune environment in two IRGPI groups. **(B)** Gene set enrichment analysis (GSEA) between two IRGPI groups. **(C,D)** Somatic mutation landscape between IRGPI low-risk and high-risk groups, respectively. **(E)** Difference of ESTIMATE immune score between two IGRPI groups. **(F)** Difference of proliferation score between two IGRPI groups.

We explored the tumor microenvironment difference by dissecting the composition of immune cells in different IRGPI groups. As shown in Figure 5A, T cells follicular helper, neutrophils, activated memory CD4 T cells, T cells gamma delta, dendritic cells resting, and M0 macrophages were more abundant in the IRGPI high-risk group, while naive B cells, resting memory CD4 T cells, and mast cells resting were more abundant in the IRGPI low-score group. The tumor microenvironment revealed that the IRGPI low-risk group reflects stronger immune response, thereby this group has better prognosis (Figures 3C,E).

The GSEA analysis was also performed to investigate the gene sets enriched in different IRGPI groups. The gene set in the IRGPI low-risk group was enriched in glycine serine and threonine metabolism, primary bile acid biosynthesis, and PPAR signaling pathway, while the gene set in the IRGPI high-risk group was enriched in cytosolic DNA pathway and immune related pathways (Figure 5B).

Somatic mutations can help to gain further insight into the tumorigenesis of HCC, thereby we plot the somatic mutation landscapes of the IRGPI low- and high-risk groups. It can be found significantly higher mutation counts in the IRGPI high-risk group than in the IRGPI low-risk group. Missense variations were the most common mutation type, followed by nonsense and frameshift deletions. We selected the top 20 genes with the highest mutation rates in two IRGPI groups (Figures 5C,D), and found the mutation rates of TP53, CTNNB1, TTN, MUC16, ALB, and PCLO were higher than 10% in both groups. The mutation of the MUC4 and LRP1B genes was more common in the IRGPI high-risk group, while the mutation of AXIN1 genes was more common in the IRGPI low-risk group.

Also, we assessed the immune infiltrating and tumor purity using ESTIMATE tool. The stromal score was considered to be correlated with the fraction of stromal cells, and the immune score reflected the infiltration of immune cells in solid tumor. As in previous studies, immune score has been confirmed to be correlated with prognosis in patients with several tumors (Yoshihara et al., 2013). In particular, Liu et al. has reported that patients with high immune scores had poor prognosis than those with low scores in HCC (Liu et al., 2020). Accordingly, we found that the immune score in IRGPI high-risk group was higher than those in IRGPI low-risk group (Figure 5E, *p* < 0.05), which is consistent with previous study.

In addition, as tumor cell proliferation leads to the overgrowth of population of clonally derived tumor cells, we explored the relationship between two IRGPI groups and tumor proliferation. As shown in Figure 5F, the cell proliferation was more active in the IRGPI high-risk group (p = 3.28e-19), and thereby leads to worse prognosis.

### 3.6. IRGPI Risk Score Closely Correlated to Immunotherapy Biomarkers

A few biomarkers have been used in clinical immunotherapy, including PD-L1/2, PD-1, CTLA-4, CYT, and TMB. Among these biomarkers, the immune checkpoint genes PD-L1/2, and CTLA4 are co-expressed in HCC (Shrestha et al., 2018). Beyond PD-L1/2 and CTLA4 level, the CYT value reflects the activity of cytotoxic T cells (CTLs) and NK cells due to their powerful ability to lyse tumor cells. A recent study found that CYT-high HCC has stronger immunogenicity and a more favorable TME than CYT-low HCC, which would result in better clinical outcomes (Takahashi et al., 2020). Also, TMB refers to the number of somatic mutations (non-synonymous mutations) that occur on an average of 1Mb base in the exon region. For immunotherapy, the higher TMB in cancer cells, the more antigens may be produced and thus stronger anti-tumor response in ICI therapy. In fact, high TMB is associated with improved response to immune checkpoint blockade in HCC (Yang et al., 2020).

**Figure 6:**
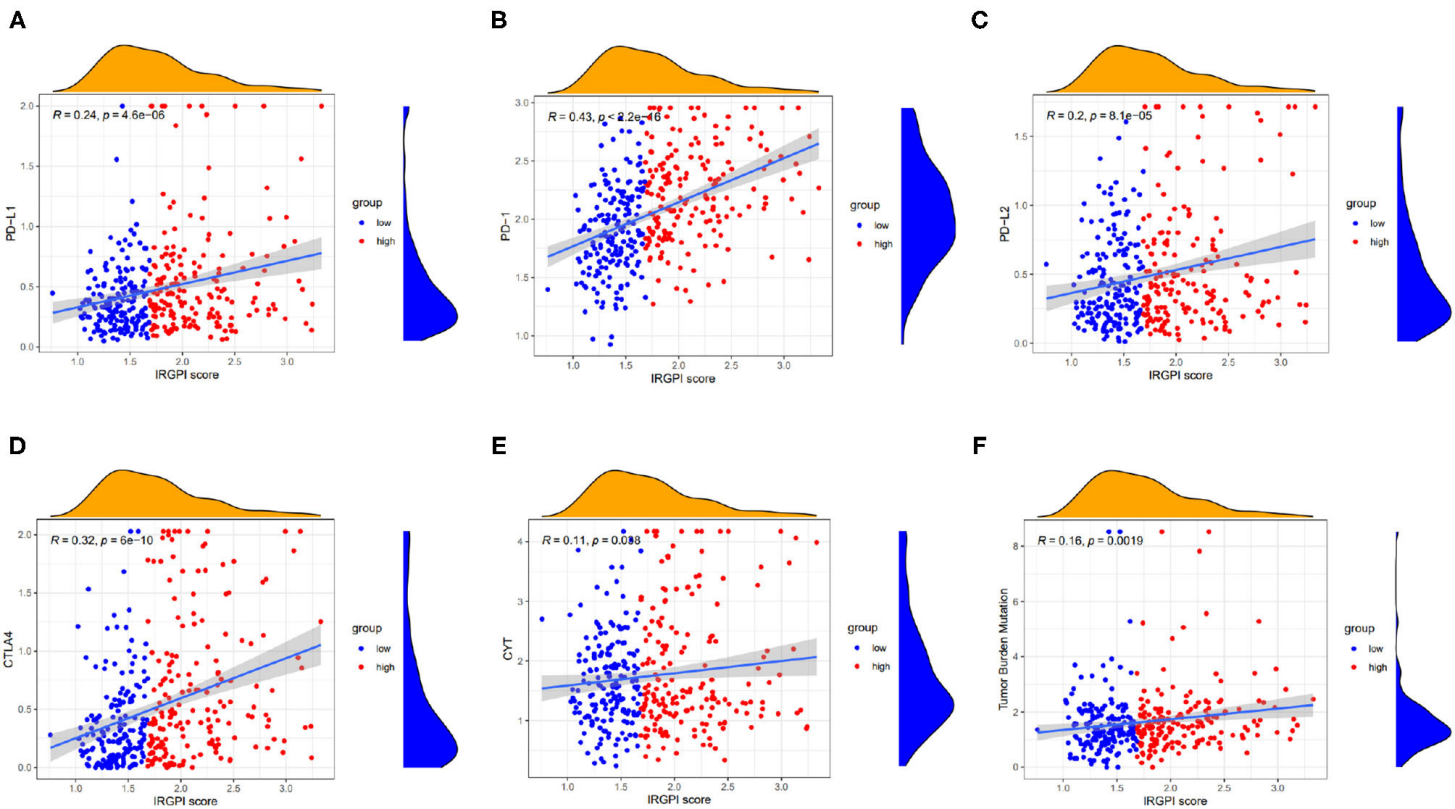
Scatter plots coordinated by IRGPI risk score and other immune biomarkers, and **(A–F)** represents PD-L1, PD-1, PD-L2, CTLA-4, CYT, and TMB, respectively.

We explored the relationship between the IRGPI scores and these biomarkers. As shown in Figures 6A–F, the IRGPI scores were positively related to the immune biomarkers. The Pearson correlation coefficients between IRGPI risk scores and PD-L1 is 0.24 with *p*-value = 4.6e-06 (PD-1: *r* = 0.43, *p* = 2.2e-16; PD-L2: *r* = 0.2; *p* = 8.1e-05; CTLA-4: *r* = 0.32, *p* = 6e-10; CYT: *r* = 0.11, *p* = 0.038; TMB: *r* = 0.16, *p* = 0.0019). The p-values of all the correlations were smaller than 0.05, suggesting that the IRGPI score is significantly correlated with immune biomarkers.

### 3.7. IRGPI Grouping Correlated to Immune Subtypes

**Figure 7:**
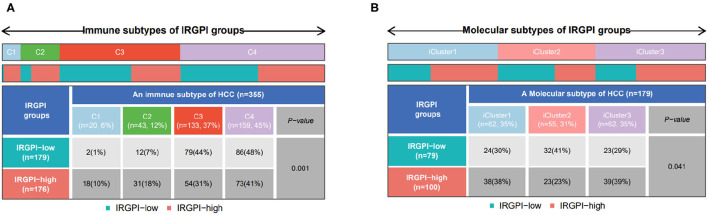
**(A)** Patient proportions between IRGPI groups among TCGA immune subtypes, and **(B)** molecular subtypes.

The immune subtypes describe the immune landscape of tumors according to the tumor and stromal compartments. A consensus immune subtyping summarized four subtypes: wound healing (C1), IFN-gamma dominant(C2), inflammatory(C3), lymphocyte depleted(C4) (Thorsson et al., 2018). Thorsson et al. have showed that C3 had the best prognosis, C2 and C1 had less favorable outcomes, while C4 conferred the least favorable outcome. Correspondingly, it can be found from Figure 7A that C3 subtype had more IRGPI low-risk patients, while other three subtypes had more IRGPI high-risk patients (*p* = 0.001, chi-square test).

Another molecular subtyping has consistently reported three immune subtypes, namely, iCluster1, iCluster2, and iCluster3 (Colaprico et al., 2016). We then focused on the distribution of the molecular subtypes in the IRGPI groups. In our study, the IRGPI low-risk group comprised 30% iCluster1 samples, 41% iCluster2 samples, 29% iCluster3 samples, while the IRGPI high-risk group comprised 38% iCluster1 samples, 23% iCluster2 samples, 39% iCluster3 samples (Figure 7B, *p* < 0.05, chi-square test). There were more samples in iCluster2 and fewer samples in iCluster1 and iCluster3 of the IRGPI low-risk group than in the IRGPI high-risk group, which is consistent with the prognosis of the molecular subtypes (Colaprico et al., 2016).

### 3.8. IRGPI Is Highly Predictive of Benefit From ICI Therapy

It has been reported that some other indicators, such as TIDE and TIS, could predict patient response to ICI therapy. TIDE (Jiang et al., 2018) is a computational method to identify factors that underlie two mechanisms of tumor immune escape. Higher TIDE score means higher potential for immune evasion, which suggested that the patients were less likely to benefit from ICI therapy and associated with worse outcome.

**Figure 8:**
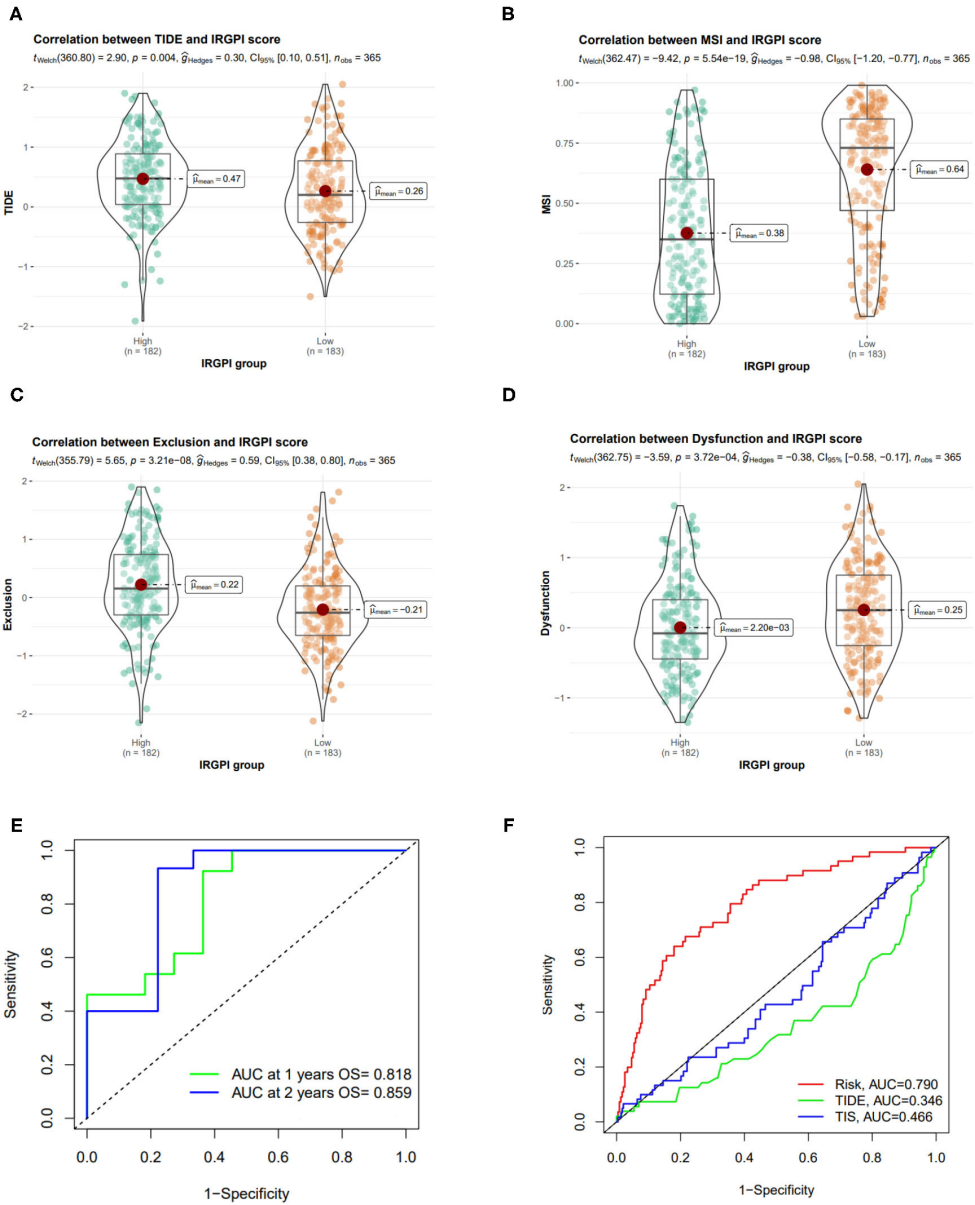
Correlation of IRGPI risk score and other immune-related prognostic scores. **(A–D)** TIDE, MSI, T cell exclusion, and T cell dysfunction score in different IRGPI groups. **(E)** ROC curve and AUC values of IRGPI risk score in predicting the 1- and 2-year OS on GSE140901 cohort who received anti-PD-L1 therapy. **(F)** Performance comparison between of IRGPI risk, TIDE and TIS in predicting 1-year OS on GSE140901 cohort.

TIDE score was used to estimate the potential clinical efficacy of ICI therapy in different IRGPI groups. The IRGPI high-risk group had higher TIDE scores than the IRGPI low-risk group (*p* = 0.004) (Figure 8A). Accordingly, we found that the IRGPI low-risk group had a higher microsatellite instability (MSI-H) score (Figure 8B), while the IRGPI high-risk group had a higher T cell exclusion score and lower T cell exclusion score in T cell dysfunction (Figures 8C,D). All these results verified that IRGPI high-risk patients would gain less benefit from ICI therapy than IRGPI-low patients.

We also tested the predictive performance of the IRGPI score on the efficacy of ICB treatment on another HCC cohort (GSE140901 dataset, *n* = 24). As showed in Figure 8E, the AUC of the ROC curve of the prognostic model reached 0.818 for 1-year OS and 0.859 for 2-year OS. Furthermore, we compare the predictive power of IRGPI with TIDE and TIS scores, and verified that the accuracy of IRGPI score was obviously higher than TIDE and TIS (Figure 8F). The results indicated that the IRGPI score might be a potential biomarker for predicting the immunotherapy response.

## 4. Discussion and Conclusion

Immune checkpoint inhibitor has been proven to be an effective treatment for HCC. Given that the overall response rate to ICI therapy remains limited, identifying patients who can benefit from ICI treatment is crucial. Since current widely used biomarkers, such as PD-L1 level, TMB, and MSI-H, have been proven to be not consistently reliable, we actually have no gold-standard biomarker for clinical ICI therapy yet. This highlights the need for an accurate prognostic biomarker for immunotherapy in HCC.

Based on the immune-related differentially expressed genes in HCC, we used WGCNA and regression analysis to identify 10 immune-related hub genes to establish a prognostic index. We computed the IRGPI score using weighted gene expression levels (see Table 1), and verified that it was an independent and effective prognostic factor. Specifically, the HCC patients with low IRGPI risk scores have improved prognosis, while those with high IRGPI risk scores have poor prognosis on TCGA cohort and two GEO cohorts. We attempted to summary the function of these genes so as to yield a mechanism explanation of the prognostic index. CARS1 is located in chromosome 11 p15.5, an important tumor-suppressor gene region. Cho et al. (2020) found that the special region of antigen-presenting cells was associated with CARS secreted by cancer cells to activate the immune response, thus stimulating a strong humoral and cellular immune response. SLC7A11 encodes a highly specific all-source of cysteine and glutamate. A few studies have shown that blocking SLC7A11 can inhibit HCC cells growth through the ROS autophagy pathway. Zhang et al. (2018) found that SLC7A11 expression profile was associated with the prognosis of liver cancer. CISD1 is an iron-containing outer mitochondrial membrane protein, and has been revealed that impaired CISD1 expression leads to tumor growth (e.g., breast and liver cancer) and has been considered a potential chemotherapeutic target (Salem et al., 2012). G6PD, a rate-limiting enzyme of the PPP, is upregulated in many cancers and contributes to tumor growth. It has been found that G6PD overexpression is significantly associated with HCC metastasis and poor prognosis of HCC (Lu et al., 2018). SLC1A5, also known as ASCT2, is one of the most studied proteins of plasma membrane transporter. Its high expression in hepatocellular carcinoma is associated with poor prognosis (Zhang et al., 2019). Consistent to these previous studies, we found these cancerogenic genes have positive weights (see Table 1) and thus contribute to high IRGPI risk score.

For in-depth understanding in terms of genetic alterations, we inspect the somatic mutation difference between IRGPI groups, and found that there was a great deal of difference in TP53 mutation. TP53 has more mutations in IRGPI-high samples than IRGPI-low samples (38 vs. 18%). TP53 is one of the most mutated genes in human cancers than any other gene, and linked with more aggressive disease and poorer patient outcomes in many cancers (Olivier et al., 2006), particularly in HCC (Hussain et al., 2007). Besides, there was a higher mutation rate occurred in MUC16 gene in the IRGPI high-risk group than IRGPI low-risk group (18 vs. 11%). It has been reported that high baseline MUC16 level is associated with poor prognosis in patients with HBV-related HCC (Qin et al., 2021). The two exemplar genes indicated that IRGPI high-risk patients bearing high TP53 and MUC16 mutations often have worse outcomes than IRGPI low-risk patients bearing low TP53 and MUC16 mutations, which is in agreement with the results of survival analysis.

TME also contributes to the difference of efficacy in immunotherapy. The composition of immune cells in tumor tissues was markedly different between two IRGPI groups. We observed that follicular helper T cells, neutrophils, resting dendritic cells, and M0 macrophages were more enriched in the IRGPI high-risk group, while the naive B cells, gamma delta T cells, resting memory CD4+ T cells were more enriched in the IRGPI low-risk group. Follicular helper T cells are perceived as a distinct CD4+ helper T-cell subset, which activates B-cell and products specific antibody responses, and acts as a basilic role in the progression of autoimmune disease. A substantial body of studies have revealed that follicular helper T cells suppress regulatory B cell development, meaning poor outcome in lung squamous cell carcinoma and gastric cancer (Zhang et al., 2017; Xu et al., 2020). In most tumors, M0 macrophages, a predominant subtype of macrophages, have been proven to be related to chronic inflammation and favor tumor growth and development of an invasive phenotype, and these cells have been associated with a negative association with prognosis (Le et al., 2021). Also, resting dendritic cell enriched in IRGPI high-risk group often leads to poor prognosis (Le et al., 2021). In contrast, gamma delta T cells are a distinct subset of T cells whose T cell receptors consist of γ chains and δ chains, regarded as a bridge between innate immunity and acquired immunity. Gamma delta T cells can not only directly kill a variety of tumor cells, but also exert indirect anti-tumor immune responses by facilitating the function of other immune cells, which suggests that gamma delta T cells may be a favorable prognostic factor (Ma et al., 2012).

For current biomarkers, such as PD-L1, TMB, and MSI-H, IRGPI risk score is significantly correlated to all of them. Other diagnostic indicator for ICI therapy, such as TIDE and TIS scores, have showed predictive performance in many solid tumors. TIDE is developed to identify factors underlying two mechanisms of tumor immune escape: the induction of T cell dysfunction in tumors with high infiltration of cytotoxic T lymphocytes (CTLs) and the prevention of T cell infiltration in tumors with low CTL levels (Jiang et al., 2018). Accordingly, IRGPI high-risk patients showed higher CTL infiltration and less T cell exclusion score (but not T cell dysfunction score), so their lower ICI response might be due to immune evasion *via* T cell exclusion. In contrast, the IRGPI low-risk patients had higher MSI scores, lower T cell exclusion score, which suggested these patients had lower levels of immune escape of HCC tumor cells.

In conclusion, our IRGPI risk score can characterize the tumor immune microenvironment, we believe it is a promising immune-related prognostic index that can predict response to ICI treatment and overall survival of HCC patients.

## Data Availability Statement

The original contributions presented in the study are included in the article/Supplementary Material, further inquiries can be directed to the corresponding author/s.

## Author Contributions

YL and HL made a comprehensive conception and analyzed the data. YL and LZ made collection of the data and implemented the analysis in a statistical manner. XL and HL gave a help to upgrade the conception and make the framework of the manuscript complete. YL was responsible for writing the draft paper. HL was responsible for revision and polished the drafts of the paper. JL and HL reinforced the supervision of the study and supplied funding support. All authors read and commented on the manuscript.

## Funding

This work was supported by National Natural Science Foundation of China (62072058; 82073339).

## Conflict of Interest

The authors declare that the research was conducted in the absence of any commercial or financial relationships that could be construed as a potential conflict of interest.

## Publisher's Note

All claims expressed in this article are solely those of the authors and do not necessarily represent those of their affiliated organizations, or those of the publisher, the editors and the reviewers. Any product that may be evaluated in this article, or claim that may be made by its manufacturer, is not guaranteed or endorsed by the publisher.
